# Monitoring and Managing Fatigue in Basketball

**DOI:** 10.3390/sports6010019

**Published:** 2018-02-27

**Authors:** Toby Edwards, Tania Spiteri, Benjamin Piggott, Joshua Bonhotal, G. Gregory Haff, Christopher Joyce

**Affiliations:** 1 School of Health Sciences, The University of Notre Dame Australia, Fremantle, WA 6959, Australia; tania.spiteri@nd.edu.au (T.S.); benjamin.piggott@nd.edu.au (B.P.); chris.joyce@nd.edu.au (C.J.); 2 Centre for Exercise and Sport Science Research, Edith Cowan University, Perth, WA 6027, Australia; g.haff@ecu.edu.au; 3 Purdue University, Purdue Sports Performance, West Lafayette, IN 47906, USA; jbonhotal@purdue.edu

**Keywords:** microtechnology, smallest worthwhile change, training load, countermovement jump

## Abstract

The sport of basketball exposes athletes to frequent high intensity movements including sprinting, jumping, accelerations, decelerations and changes of direction during training and competition which can lead to acute and accumulated chronic fatigue. Fatigue may affect the ability of the athlete to perform over the course of a lengthy season. The ability of practitioners to quantify the workload and subsequent fatigue in basketball athletes in order to monitor and manage fatigue levels may be beneficial in maintaining high levels of performance and preventing unfavorable physical and physiological training adaptations. There is currently limited research quantifying training or competition workload outside of time motion analysis in basketball. In addition, systematic research investigating methods to monitor and manage athlete fatigue in basketball throughout a season is scarce. To effectively optimize and maintain peak training and playing performance throughout a basketball season, potential workload and fatigue monitoring strategies need to be discussed.

## 1. Introduction

Basketball is an intermittent, court-based team sport comprised of repeated high intensity movements such as change of direction, accelerations and decelerations interspersed with periods of low to moderate intensity activity [1]. Athletes also perform regular maximal efforts during competition including extensive high intensity shuffling, sprinting and jumping [2,3]. Research using time motion analysis (TMA) to investigate the competition demands of basketball have revealed that the mean distance covered by female and male athletes was 5–6 km during live playing time across 40 min games [1]. Physiological traits such as blood lactate and heart rate responses to competition demands reveal athletes are competing at an average physiological intensity above lactate threshold and 85% maximum heart rate [1]. The competition demands encountered by basketball players suggest that both anaerobic and aerobic energy pathways contribute to energy sources. Basketball also has one of the longest seasons in professional sports. Typically, a professional National Basketball Association (NBA) season consists of 82 games played over six months. If successful, teams can play over 100 games if they make post season play offs. Competitive seasons in Division I collegiate basketball in the United States span five months and include approximately 30 regular season games which is consistent with other semi-professional and professional leagues around the world. The high intensity movement demands and physiological stress on the athletes during competition may accumulate over the pre-season and competitive season and present as signs of fatigue leading to decreased performance output and/or injury [4]. Combining objective and subjective measures of workload and fatigue provides practitioners such as strength and conditioning coaches and sport scientists with a global picture of how the athlete is responding to the training dose, competition demands and non-training stressors. Early identification and subsequent management of fatigue may prevent detrimental physical and physiological adaptations often associated with injury and enhance athletic performance and player availability [5,6].

**Figure 1:**
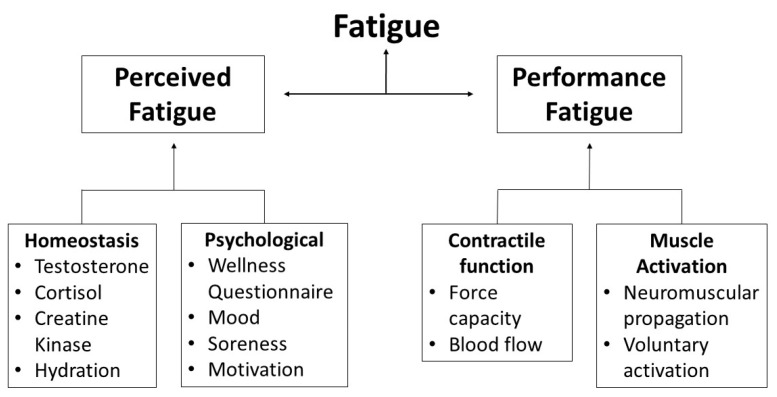
Modulating factors of perceived and performance fatigability (Adapted from [11]).

It is well understood that fatigue can inhibit athletic performance [4,7,8,9,10], however, conflicting definitions of fatigue make monitoring and measuring the underlying fatigue mechanisms problematic. The two attributes of fatigue that need to be acknowledged are: perceived fatigability, the maintenance of homeostasis and subjective psychological state of the athlete; and performance fatigability, the decline in objective performance measures derived from the capacity of the nervous system and contractile properties of muscles over time [11] (Figure 1). To align with a recent report [11], this review will define fatigue as a symptom where cognitive and physical function is limited by the interaction between perceived fatigability and performance fatigability. These two measures are able to normalize observed fatigue to the demands associated with the sport. For example, athletes who are less fatigable are able to endure a greater amount of workload before reaching a given level of fatigue [11]. Whilst multiple methods to monitor and manage perceived and performance fatigue in other sports have been investigated such as sprint speed [12,13], wellness questionnaires [14,15,16], biochemical markers [17] and neuromuscular tests [18], there is a lack of research examining the longitudinal use of these methods and practices in basketball. This review aims to provide practitioners with an overview of fatigue monitoring tools and management methods that have been reported in the literature or appear suitable in collegiate or professional basketball. 

## 2. Materials and Methods

The search strategy used to locate articles included an online search of journal databases including PubMed, Web of Science, EBSCO host and Google Scholar. Key terms used in the search included monitoring OR managing AND fatigue OR performance AND basketball. In addition, articles cited in the reference lists of identified journals were manually searched and examined.

## 3. Quantifying Workload

Before managing fatigue, it is important to quantify and understand the training and competition workload the athlete has completed. Combining the athletes’ workload and fatigue measurement will allow practitioners to determine the dose-response relationship and help inform whether the athlete is prepared for competition. Since the physical and physiological adaptations from a training stimulus vary between individuals based on modifiable (strength, aerobic/anaerobic capacity) and non-modifiable (age, gender, anatomy, genetics) factors, it is necessary to monitor the individual dose-response relationship [6]. For example, a strong correlation was detected in elite European basketball athletes between distance covered in the Yo-Yo intermittent recovery level one test and session rating of perceived exertion (s-RPE) scores during practice (r = 0.68) [19]. This suggests that assuming athletes achieved equal amount of workload, athletes with an increased aerobic capacity perceive the same training session as being easier than athletes who have a lower aerobic capacity. Currently, TMA and s-RPE are the most common methods to quantify the movement and workload demands in basketball [20], however recent advances in technology have allowed microtechnology devices to objectively quantify the external load of athletes in training and competition. A recent review of player monitoring approaches in basketball extensively discusses the advantages and disadvantages of several methods to quantify an athlete’s workload [20]. A unique aspect of this review is that it adds to Fox and colleagues [20] review by briefly discussing the findings and results of previous research that has investigated basketball training and/or competition demands using microtechnology or s-RPE.

### 3.1. Microtechnology

Microtechnology has become a popular tool for practitioners and researchers to monitor and quantify the physical demands of athletes during training and competition in outdoor field sports such as soccer, rugby league, rugby union and Australian Rules football (AF) [21,22]. However, quantifying the external demands of basketball using microtechnology is challenging due to several limitations including that the game is played in indoor stadiums, the feasibility of acquiring enough units and the reliability and validity of microtechnology to detect basketball specific movements [22]. Recently, advances in technology have integrated a number of micro inertial sensors including triaxial accelerometers, gyroscopes and magnetometers into single units commonly referred to as inertial measurement units (IMU) [22]. These devices have assisted in overcoming some of the previously mentioned limitations that surround quantifying movement demands in basketball training and competition. The IMU provides an array of information that can inform practitioners’ decisions on the performance of basketball players in training and competition including the position, direction, velocity, accelerations and decelerations [21,22]. A recent study used a tri-axial accelerometer with a sample rate of 100 Hz to examine the external demands of common training drills [23]. Instantaneous data from all 3 axes (x, y and z) were assimilated into a resultant vector through the Cartesian formula √[(x_n_ − x_n−1_)^2^ + (y_n_ − y_n−1_)^2^ + (z_n_ − z_n−1_)^2^]. Accelerometer Load (AL) for each drill and activity was then calculated by summating the instantaneous change of rates of resultant accelerations over time [23]. The authors reported full court 3v3 and 5v5 (18.7 ± 4.1; 17.9 ± 4.6 AL/min, respectively) produced greater AL than full court 2v2 and 4v4 (14.6 ± 2.8; 13.8 ± 2.5 AL/min, respectively) [23]. In regards to playing position, the authors reported higher AL for point guards irrespective of training drill. This may represent tactical requirements of the position as smaller players may be required to cover more distance per possession. Another logical reason for guards to have greater AL is that they are able to accelerate easier with less applied force due to lower body mass [23]. A more recent investigation into training and competition demands of semi-professional basketball players reported significantly higher absolute and relative AL during game based training than competition (624 ± 113 AL vs. 449 ± 118 AL, ES = 1.54; 6.10 ± 0.77 AL/min vs. 4.35 ± 1.09 AL/min, ES = 2.14 respectively) [24]. This shows that pre-season training in semi-professional basketball appears to adequately prepare players for competition. The combination of these findings, and the application of IMUs in basketball, may help practitioners improve athletes’ conditioning by developing position specific drills, improve training periodization, and provide more accurate drill clarification and description. However, systematic monitoring of external demands using IMUs is still warranted to provide a greater understanding of the suitability and effectiveness of the devices in basketball to quantify basketball activities such as shuffling and jumping. 

### 3.2. Session Rating of Perceived Exertion

An issue that practitioners are commonly faced with when quantifying an athlete’s workload is that the different scale, units and type vary across different training modalities. For example, comparing the load of a resistance training session (sets × reps × weight) and a court based training session (accelerations, decelerations, velocity) is problematic as there is no single objective load monitoring variable for both modalities of training. The simple method of s-RPE can overcome this issue and be used across several training modalities to monitor an athlete’s perceived exertion from a particular training session [25,26] and longitudinally across an entire season [27,28]. By using a modified Borg RPE scale ranging from 0 to 10 which represent rest and maximal exertion respectively, athletes can provide a subjective rating of the intensity of a particular training session [26]. This number is multiplied by the duration in minutes to provide an arbitrary unit of subjective internal training load [26]. Unlike microtechnology, s-RPE has been widely reported in basketball literature that has investigated internal responses to training and competition [3,19,29,30,31,32]. In elite European basketball weekly s-RPE training load significantly differed between the control week (no game) and those accumulated during 1 or 2 game week microcycles (3334 ± 256 vs. 2928 ± 303 vs. 2791 ± 239 arbitrary units (AU), respectively) [19]. In addition, authors reported a strong correlation between s-RPE and heart rate based training load model (Edwards’ TRIMP) in the same population (r = 0.68) [19]. However, s-RPE exhibited a moderate relationship (r = 0.49) and low commonality (R^2^ = 0.24) with accelerometer derived training load in semi-professional Australian basketball players [32]. This suggests that s-RPE measures different training constructs than external AL. Therefore, it is recommended that practitioners collect both external and internal training load measures such as s-RPE and accelerometer or IMU training load as the intermittent demands and lateral movements required in basketball can increase an athlete’s s-RPE by 13–25% when external load is controlled [33]. S-RPE is non-invasive, simple to calculate and quantify across the length of a basketball season making it an efficient and practical tool to use in both research and practice. 

## 4. Fatigue Monitoring Tools

A number of different fatigue monitoring tools exist that may assist practitioners in identifying indicators of performance and perceived fatigability in basketball athletes including sprinting ability, vertical jumps, athlete self-report measures (ASRM), heart rate indices and biochemical markers. These fatigue monitoring tools may be beneficial in monitoring athletes’ fatigue levels during a long season where accumulation of fatigue may affect player on court performance. Incorporating several fatigue monitoring tools simultaneously may provide practitioners with a global understanding of how athletes are responding to training and non-training stressors. Subsequently, a player’s prescribed workload can be altered as necessary. 

### 4.1. Sprinting Ability

Sprinting is a critical movement performed by all players during basketball training and competition [34]. Sprint speed has been identified as an important attribute of basketball athletes, specifically 5 m sprint times has exhibited a moderate inverse relationship to playing time (r = −0.59) in the NCAA Division II competition [35]. Conversely, 20 m sprint time demonstrated a weak correlation to total playing time [35,36] and basketball specific statistics including points, assists, rebounds, steals and blocks [37]. Monitoring an athlete’s acceleration ability may be a more appropriate method to identify fatigue in basketball athletes in contrast to maximal speed as players rarely sprint the length of the court and therefore do not reach maximal speed in competition [34]. However, conjecture surrounds the use of sprint assessments as fatigue monitoring tools in previous literature. In rugby league, non-significant changes in 10 and 40 m were reported following six weeks of deliberate overreaching [28]. Meanwhile, in soccer players, 20 yard (18.3 m) sprint times decreased in starters and not in non-starters during 11 weeks of soccer competition [13]. Specifically, to basketball 10 m sprint time was decreased up until 24 h post-match (ES = 0.5) in elite European basketball players [38]. Therefore, monitoring acceleration ability over 5 to 10 m seems promising as a measure of performance fatigability in basketball. 

### 4.2. Athlete Self-Report Measures

A recent survey on fatigue monitoring tools in high performance sport reported a high usage of ASRM across various sports and levels of competition for assessing overall well-being of team sport athletes [4]. Several ASRM have been used in the literature including the Profile of Athlete Mood States (POMS) [39], Daily Analysis of Life Demands of Athletes (DALDA) [40], Total Quality Recovery (TQR) [41] and the Recovery Stress Questionnaire for Athletes (REST-Q) [28]. However, to minimize time constraints on athletes, many team sport practitioners prefer shorter, customized versions that can be completed on a daily basis [4]. The shorter customized ASRM has been shown to be sensitive to daily, weekly and seasonal changes in training load in elite AF and English soccer players [14,16,42]. Specifically, daily ASRM that included fatigue, sleep quality, stress, mood and muscle soreness significantly associated with daily fluctuations in training load during the pre-season and competitive periods of elite AF and English soccer players respectively [16,42]. More recently, pre-training subjective ASRM have been suggested to provide practitioners with information on an athlete’s capacity to train [7,15]. For example, in American collegiate football an increase of one unit in muscle soreness (players felt less sore) *z* score led to a trivial 4.4% decrease in s-RPE training load [7]. In AF, a one unit decrease in wellness *z* score corresponded to 4.9% decrease in player load [15]. The *z* score indicates how many standard deviations a variable is from the mean and can be calculated using the following formula: *z* score = athlete’s score—athlete’s mean score/standard deviation (SD) of athlete’s score [7,8,15]. Whilst there is limited research investigating customized ASRM in basketball, the evidence in several other sports suggest that lower pre-training wellness scores may lead to a decrease in external load and an increase in internal load [7,9,15,40]. Implementing daily ASRM into an athlete monitoring program for basketball athletes may assist practitioners in understanding the perceptual fatigue of athletes, how they are coping with training and competition schedule, and also provide insight into intensity of output expected from an athlete in training. 

### 4.3. Vertical Jumps 

The use of vertical jump performance as a fatigue monitoring tool is also popular in high performance sport to assess lower body strength and power, and the integrity of the musculotendinous pre-stretch, or countermovement stretch shortening cycle (SSC) [43,44]. More than half of the respondents (54%) in a fatigue monitoring survey reported using vertical jump testing on either a daily, weekly or monthly basis to monitor performance and neuromuscular fatigue [4]. A variety of offensive and defensive movements are completed by basketball athletes during training and competition including accelerating, decelerating and change of direction that rely heavily on the athletes ability to rapidly transition from eccentric to concentric contraction via the SSC [45]. Repetitive performance of these movements can result in reduced movement efficiency through neuromuscular and performance fatigue [43,44]. Several vertical jump protocols have been used to monitor neuromuscular function and the SSC including the drop jump (DJ) and the countermovement jump (CMJ) [4,9]. In addition, a number of different apparatus have also been used in the literature to monitor vertical jump performance in athletes including a Vertec system (jump and reach) [12], contact mats [46], force plates [18] and linear position transducers [45]. Many of these protocols and instruments can be administered, analyzed and reported quickly in order to make decisions regarding the athlete’s daily or weekly training prescription. 

A meta-analysis reported using the average height of multiple CMJs was more sensitive in detecting CMJ fatigue and supercompensation than the maximum CMJ height [47]. However, conflicting evidence surrounds the use of jump height as the sole fatigue monitoring variable. For example, results from a 3 day elite handball competition demonstrated significant decline in CMJ height [48] though no changes in CMJ height were observed in elite rugby sevens players during the final preparation period [49]. The inconsistent findings surrounding jump height as a global indicator of neuromuscular function and performance fatigue is likely due to its gross representation of several underlying kinematic variables that contribute to CMJ height. These underlying variables that contribute to CMJ height relate to the eccentric and/or concentric phase and may provide a greater insight into the integrity of the SSC, loading strategies and behaviors used to execute a CMJ [50]. Findings from a study investigating the response of a CMJ following training and competition suggest flight time to contraction time (FT:CT) ratio appears to be a sensitive measure able to detect neuromuscular fatigue in female basketball athletes [51]. In contrast, basketball players reactive strength index (flight time/contact time) derived from a 40 cm drop jump was not sensitive to detect changes in s-RPE training load during a competitive elite Australian basketball season [46]. It is difficult to make comparisons between the two findings as they both elicit different loading strategies and behaviors. For example, the CMJ assesses a slow SSC response (contact time >250 ms) whilst the DJ elicits a fast SSC response (contact time <250 ms) [46]. Despite this, administering vertical jump performance test as fatigue monitoring tools seem promising as high levels of neuromuscular function is critical to vertical jumping capacity, change of direction ability and basketball performance [52]. 

### 4.4. Heart Rate 

The autonomic nervous system (ANS) is linked with many physiological systems and can potentially identify fatigue and negative training adaptations through alterations in heart rate [53]. Specifically, several heart rate derived metrics including resting heart rate (RHR) and heart rate variability (HRV) have the potential to provide practitioners with an understanding of how an athlete is responding to fluctuations in training and competition workload. The use of heart rate metrics for monitoring athlete fatigue has been comprehensively reviewed [53], therefore the following will provide a brief overview of each variable and the applicability to basketball. 

#### 4.4.1. Resting Heart Rate

One of the first signs of overtraining syndrome commonly reported in the literature is an increase in RHR [54]. However conflicting research exists with some early investigations reporting increased RHR in overreaching athletes and those with overtraining syndrome [55], whilst other studies found RHR remained similar in overreaching and normal states [56,57]. A systematic review of 34 studies investigated whether RHR can be used to determine overreaching in athletes reported moderate increase in RHR after short (<2 weeks) interventions but no difference was found in longer (>2 weeks) interventions [54]. These findings suggest that the use of RHR to monitor fatigue in basketball athletes may be beneficial during intensive training camps (<2 weeks) and congested fixtures where spikes in workload are common potentially leading to an increase in fatigue. Consequently, including RHR in a longitudinal athlete monitoring system over the length of a season or to monitor non-functional overreaching or over training syndrome may not provide a valid sign of fatigue. 

#### 4.4.2. Heart Rate Variability

Research investigating changes in HRV in athletes during heavy training and competition periods has received increased interest due to the high reliability and the ability to capture data over a short period (~60 s) [58,59]. A common interval period often used as an index of ANS responsiveness is known as the R-R interval, or the time between heart beats. Whilst RHR can remain relatively stable, vagal related time periods can vary substantially [59]. However, conflicting findings are reported in the literature in relation to the use of HRV as a fatigue monitoring tool. Specifically, HRV has demonstrated sensitivity to changes in workload and performance in individual sports such as weightlifting [60], swimming [61] and middle-distance running [62] with only trivial evidence in team sport athletes [9,10]. In spite of the support of HRV in individual athletes, a systematic review reported only small effects of overreaching on HRV [54]. Similarly to RHR, this finding was also limited to short (2 weeks) interventions/overload [54]. An absence of research in determining the use of HRV in basketball athletes suggest that more research is needed to further clarify its usefulness as a fatigue monitoring tool. In addition, previous research in team sports suggest using caution if including HRV in an athlete monitoring program [9,10,54]. 

### 4.5. Biochemical Markers 

When prescribing an athlete’s workload to optimize training adaptations and avoid inducing further fatigue, it is important to remember that the endocrine system plays an important role [63]. The most commonly investigated biochemical markers in response to workload are testosterone and cortisol. Testosterone is an anabolic hormone that promotes amino acid incorporation into proteins whilst inhibiting protein breakdown [63]. Approximately 98% of testosterone is bound to carrier proteins such as sex-hormone-binding globulin (54%) and albumin and other proteins (44%) [64]. Of importance to practitioners is free testosterone, which is the part of serum testosterone that is available to tissues of the body [64]. Monitoring free testosterone levels can provide practitioners with an understanding of the anabolic status of the body [63]. Greater levels of free testosterone have been seen as a result of acute heavy resistance training [63]. However, conflicting findings have been reported in regards to the effect of training volume on resting free testosterone levels. A short-term investigation found a negative correlation between resting free testosterone levels with increases in training volume [65], whilst longitudinal studies have reported no changes in resting levels [66].

Cortisol is a catabolic hormone that converts amino acids to carbohydrates when muscle glycogen levels are depleted [63]. Similar to testosterone, there have been varied reports on the acute response of cortisol to workload with cortisol levels returning to pre-exercise levels within 2 to 3 h after cessation of exercise [67] whilst increased levels have also been observed for up to 24 h [67]. The free testosterone:cortisol (TC) ratio represents the imbalance between anabolic and catabolic state of the athlete or response to workload and has been used as a marker to determine anabolic and catabolic activity during periods of increased workloads [66,68,69]. During an 11-week training period in female weightlifters a very strong relationship was reported between percentage change in TC ratio and volume load (r = −0.83) and training intensity (r = −0.72) suggesting concomitant changes in the anabolic to catabolic ratio [17]. Specifically in regards to athlete monitoring, a decrease of 30% has been attributed towards overtraining with a number of investigations reporting significant relationships between performance and the TC ratio [8,66,69]. A longitudinal study involving basketball athletes over four consecutive years concluded that an athlete’s hormonal status is linked with playing position, with power forwards and small forwards exhibiting the most catabolic state [70]. Overall, all players presented the most catabolic state in the final third of the regular season [70]. However, no changes were noted in testosterone, cortisol or TC ratio during a 28 day training camp in elite basketball athletes [71]. 

In addition to testosterone and cortisol, creatine kinase (CK) is also a commonly measured fatigue marker in athletes. The CK enzyme is stored inside muscle cells, however after heavy exercise is often released into the blood reflecting muscle damage [8]. Hence practitioners are interested in measuring CK levels to determine the level of exercise induced muscle damage. Acute CK responses have been documented in basketball [72] with increases following three days of tournament play. Longer investigations have also demonstrated increases in CK levels in team sport and non-team sport athletes. For example, following a six week deliberate overreaching phase in rugby league players, a significant increase in CK levels was observed [73]. Similar results were reported during six weeks of progressive endurance training in healthy adults [74]. The above evidence appears appealing for use of CK as a fatigue monitoring tool in basketball; however, large individual variability in resting CK levels exist which can make it problematic to measure change induced by training [75]. It is recommended that practitioners establish baseline levels for each athlete from a large number of samples in order to understand the degree of variability [8].

## 5. Fatigue Management

The advantages and disadvantages of the fatigue monitoring tools discussed in this review are outlined in Table 1 however practitioners can face unique challenges and scenarios depending on the time of year [8]. For example athletes are required to complete higher training volumes during a training camp or pre-season period. Strength and conditioning practitioners may use different strategies to manage athlete fatigue during this period compared to the competition period. The following sections discuss several methods in which the practitioner can manage athlete fatigue during intensive training camps and the competition period.

### 5.1. Training Camp

Typically training camps or pre-season can last from seven weeks in collegiate basketball to only three weeks in the NBA. During this period athletes are exposed to high training loads to physically prepare them for the upcoming season. A challenge that faces strength and conditioning practitioners and coaching staff is the prescription of appropriate training volumes and recovery periods to optimize physiological adaptation and development of technical and tactical skills without the negative effects of high training loads [8]. Research indicates that players who complete a greater number of training sessions in the pre-season have a reduced injury rate during the competitive season [76]. Evidence also shows that teams with the lowest injury burdens had greater success in competition [77]. Whilst basketball training camps are typically shorter than those of other sports such as AF and rugby, it is recommended that strength and conditioning practitioners avoid large spikes (>10%) in workload to avoid increased risk of injury [5]. Given the shortened training camp in basketball compared to other sports, it is important to assess prior training load history as the off season break generally results in a low training base or chronic workload. A multi-disciplinary approach between sport coaches, strength and conditioning coaches, sport scientists, and athletes may be able to reduce the large spikes in workload period by voluntarily completing more training prior to the training camp, less training at the camp or a combination of both to ensure individual training prescription [5]. In addition, pairing individual athletes workload with fatigue monitoring tools will provide a global understanding of the dose-relationship and how the athlete is coping with the current workload [8]. Research studies report that no single fatigue monitoring tool can give a complete picture of an athlete’s response to training and recommend using several fatigue monitoring tools across the squad of athletes to inform training and recovery decisions [14]. An example athlete monitoring system for training camp in basketball is detailed in Table 2. 

### 5.2. Competition Periods

A concern for practitioners during the competition period of basketball season is the impact of travel and the different turnaround times between matches. This must be considered by practitioners and sport coaches when planning the team’s training program. Evidence in rugby league demonstrated that some positions had higher injury rates with longer turnaround times, whilst those in other positions had higher injury rates in shorter turnaround times [78]. Whilst there is limited research in basketball investigating the effects of travel and different turnaround times, practitioners need to take the positional differences into account as physical demands of training and competition are largely varied [23]. Quantifying individual athlete’s workload and fatigue response can provide practitioners with insight in to how each athlete responds to travel, turnaround times and match load [8,78]. 

In any given week collegiate basketball teams play two games whilst in the NBA teams can play up to five games in a seven day period. Congested fixtures are also prevalent in post-season tournament play in which athletes are required to compete with only 24 h between games [8]. Evidence suggests higher levels of fatigue and increased injury rates are associated with congested fixtures due to spikes in game load [79]. Specifically, to basketball, 10 m sprint speed and CMJ height decreased until 24 (ES = 0.5) and 48 h (ES = 0.6) post-match [38]. These findings indicate that basketball athletes may need ~24–48 h of recovery post-match before the next intensive practice or match. Implementing fatigue monitoring strategies into daily training sessions such as CMJ and wellness questionnaires in combination with internal and external workload monitoring tools may assist practitioners to inform training and recovery strategies [8,38]. Longitudinally, athlete monitoring systems provide strength and conditioning practitioners a clearer understanding of scheduling variations and how each athlete responds to certain situations. 

## 6. Interpretation Considerations

Before basketball practitioners use a fatigue monitoring tool it is important to ascertain the tool’s reliability within their population as it has been shown to differ between sports and competition levels [8]. Several methods of assessing reliability of monitoring tools exist however intraclass correlations (ICC) and coefficient of variation (CV) are most common [8]. An ICC is used to determine the relationship between repeated tests or monitoring tools. A correlation of 1.0 represents a perfect relationship whilst 0.0 represents no relationship [8]. The CV refers to the typical error of a variable expressed as a percentage of the athlete’s mean. A variable is often considered reliable when the ICC is >0.8 and/or if the CV is <10% [9]. In addition to establishing the reliability of a particular variable, the smallest worthwhile change (SWC) should also be calculated to allow practitioners to determine the smallest practical change in a fatigue monitoring tool that is important or worthwhile [8]. To calculate the SWC the following formula can be used: 0.2× between-subject standard deviation [8]. The SWC should be put into the context of the reliability of the fatigue monitoring tool. For example, for a practitioner to be confident that a change is not due to the noise associated with the test, the SWC should be greater than the CV [8]. However, it is important to incorporate both the CV and SWC together to determine the reliability and sensitivity of a fatigue monitoring tool variables that express the highest reliability may be too consistent and not sensitive to changes in athletic performance [8]. In contrast, variables that express poor reliability may be sensitive to fatigue despite having large variations that are greater than the SWC [8]. Therefore it is necessary that practitioners establishing the above statistics within their population in order to identify when athletes are in a fatigued state. 

## 7. Conclusions

Basketball athletes playing at a professional or collegiate level participate in demanding pre-seasons to prepare for long playing seasons often coupled with extensive travel schedules. Ultimately this may result in an accumulation of perceptual and/or performance fatigue which could lead to a decrease in playing performance. Therefore, it is important that sport scientists and strength and conditioning practitioners implement appropriate athlete monitoring protocols to: (1) monitor the activity demands of training and competition; (2) monitor athlete fatigue levels; (3) prescribe appropriate recovery sessions; and (4) subsequently adjust and manage the athletes’ workloads in order to potentially decrease and prevent high levels of fatigue that may affect playing performance. This review discussed several methods that may be used to quantify workload and athlete fatigue in basketball. However, it is important when practitioners pursue workload and fatigue monitoring tools that they also consider the feasibility, applicability and availability of equipment or resources.

Implementing an athlete monitoring program that includes workload and fatigue monitoring and management in basketball may assist practitioners and sport coaches to prescribe appropriate workloads that optimize training adaptations, decrease accumulated fatigue and allow athletes to perform at their highest level. Many of the discussed workload and fatigue monitoring tools have not been longitudinally investigated in basketball but are supported in several other sporting populations. Caution should be taken when initially implementing them into an athlete monitoring program. However, by implementing several workload and fatigue monitoring methods simultaneously, valuable information on an athlete’s global fatigue and general workload trends over the length of a basketball season can be further understood. It should be noted that this review discusses common fatigue monitoring tools and variables reported in the literature that may be applied to monitor workload and fatigue in basketball. Several other methods can also be applied and incorporated in to an athlete monitoring program that may effectively monitor fatigue in basketball players that should also be investigated. 

## Figures and Tables

**Table 1:** Advantages and Disadvantages of Fatigue Monitoring Tools.

Fatigue Monitoring Tool	Advantages	Disadvantages
**Vertical Jumps**	Easy to administer Minimal additional fatigue Replicates common athletic movement performed in competition Easily implemented	Lack of motivation to perform maximally No consensus as to which variable is most sensitive to fatigue Limited information regarding cause of performance reduction
**Wellness Questionnaire**	No additional fatigue Can be completed on a daily basis Easy to administer	Rely on subjective information Athletes can manipulate data
**Sprint Assessment**	Replicates movement performed in competition Easily implemented Provides information even when athlete not in a fatigue state	May add to existing fatigue Lack of motivation to perform maximally Limited information regarding cause of performance reduction
**Resting Heart Rate + Heart Rate Variability**	Most accessible physiological measure Ability to capture over short period of time	Valid for short term (<2 weeks) overload only Limited evidence support use in team sports
**Biochemical Markers**	Assist in understanding whether athlete is in a catabolic or anabolic state CK levels may help determine level of muscle damage	High time, cost and expertise demand for data collection Time consuming analysis and feedback

**Table 2:** Example Monitoring System for a Basketball Training Camp [8].

Monitoring Tool	Frequency	Purpose	Analysis Method	Interpretation
**Microtechnology (Player Load)**	Every court based session	Measure of external load	*Z*-score relative to individual Acute to chronic ratio	*Z*-score ≤ −1.5 Acute to chronic ratio ≥1.5 = increased risk of injury
**S-RPE training load**	Every session	Measure of internal load	*Z*-score relative to individual Acute to chronic ratio	*Z*-score ≤ −1.5 Acute to chronic ratio ≥ 1.5 = increased risk of injury
**Wellness Questionnaire**	Daily	Measure of sleep quality, fatigue, soreness etc.	*Z*-score to baseline measures Smallest meaningful change relative to reliability	*Z*-score ≤ −1.5 ± on item = positive or negative change
**Countermovement Jump**	Daily	Measure of neuromuscular fatigue	*Z*-score to baseline measures Smallest meaningful change relative to reliability	*Z*-score ≤ −1.5 If a variable decreases greater than the SWC
**RHR/HRV**	Daily	Measure of ANS	*Z*-score to baseline measures	*Z*-score ≤ −1.5

RHR = Resting Heart Rate; HRV = Heart Rate Variability; ANS = Autonomic Nervous System; S-RPE = Session Rating of Perceived Exertion; SWC = Smallest Worthwhile Change.

## References

[1] Stojanović E., Stojilijković N., Scanlan A.T., Dalbo V.J., Berkelmans D.M., Milanović Z. (2017). The activity demands and physiological responses encountered during basketball match-play: A systematic review. Sports Med..

[2] Abdelkrim N.B., El Fazaa S., El Ati J. (2007). Time-motion analysis and physiological data of elite under-19-year-old basketball players during competition. Br. J. Sports Med..

[3] Scanlan A., Dascombe B., Reaburn P. (2011). A comparison of the activity demands of elite and sub-elite Australian men’s basketball competition. J. Sport Sci..

[4] Taylor K., Chapman D., Cronin J., Newton M.J., Gill N. (2012). Fatigue monitoring in high performance sport: A survey of current trends. J. Aust. Strength Cond..

[5] Drew M.K., Cook J., Finch C.F. (2016). Sports-related workload and injury risk: Simply knowing the risks will not prevent injuries. Br. J. Sports Med..

[6] Windt J., Gabbett T.J. (2016). How do training and competition workloads relate to injury? The workload—injury aetiology model. Br. J. Sports Med..

[7] Govus A.D., Coutts A., Duffield R., Murray A., Fullagar H. (2017). Relationship between pre-training subjective wellness measures, player load and rating of perceived exertion training load in American college football. Int. J. Sports Physiol. Perform..

[8] McGuigan M. (2017). Monitoring Training and Performance in Athletes.

[9] Thorpe R.T., Atkinson G., Drust B., Gregson W. (2017). Monitoring fatigue status in elite team sport athletes: Implications for practice. Int. J. Sports Physiol. Perform..

[10] Thorpe R.T., Strudwick A.J., Buchheit M., Atkinson G., Drust B., Gregson W. (2016). Tracking morning fatigue status across in-season training weeks in elite soccer players. Int. J. Sports Physiol Perform..

[11] Enoka R.M., Duchateau J. (2016). Translating fatigue to human performance. Med. Sci. Sports Exerc..

[12] Coutts A.J., Reaburn P., Piva T.J., Rowsell G.J. (2007). Monitoring for overreaching in rugby league players. Eur. J. Appl. Physiol..

[13] Kraemer W.J., French D.N., Paxton N.J., Volek J.S., Sebastianelli W.J., Putukian M., Newton R.U., Rubin M.R., Gomez A.J., Vascovi J.D. (2004). Changes in exercise performance and hormonal concentrations over a big ten soccer season in starters and nonstarters. J. Strength Cond. Res..

[14] Buchheit M., Racinais S., Bilsborough J.C., Bourdon P.C., Voss S.C., Hocking J., Cordy J., Mendez-Villanueva A., Coutts A.J. (2013). Monitoring fitness, fatigue and running performance during a pre-season training camp in elite football players. J. Sci. Med. Sport..

[15] Gallo T.F., Cormack S.J., Gabbett T.J., Lorenzen C.H. (2016). Pre-training perceived wellness impacts training output in Australian football players. J. Sports Sci..

[16] Gastin P.B., Meyer D., Robinson D. (2013). Perceptions of wellness to monitor adaptive responses to training and competition in elite Australian football. J. Strength Cond. Res..

[17] Haff G.G., Jackson J.R., Kawamori N., Carlock J.M., Hartman M.J., Kilgore J.L., Morris R.T., Ramsey M.W., Sands W.A., Stone M.H. (2008). Force-time curve characteristics and hormonal alterations during an eleven-week training period in elite women weightlifters. J. Strength Cond. Res..

[18] Cormack S.J., Newton R.U., McGuigan M.R., Cormie P. (2008). Neuromuscular and endocrine responses of elite players during an Australian rules football season. Int. J. Sports Physiol. Perform..

[19] Manzi V., D’ottavio S., Impellizzeri F.M., Chaouachi A., Chamari K., Castagna C. (2010). Profile of weekly training load in elite male professional basketball players. J. Strength Cond. Res..

[20] Fox J.L., Scanlan A.T., Stanton R. (2017). A review of player monitoring approaches in basketball: Current trends and future directions. J. Strength Cond. Res..

[21] Cummins C., Orr R., O’Connor H., West C. (2013). Global positioning systems (GPS) and microtechnology sensors in team sports: a systematic review. Sports Med..

[22] Malone J.J., Lovell R., Varley M.C., Coutts A.J. (2016). Unpacking the black box: Applications and considerations for using GPS devices in sport. Int. J. Sports Physiol. Perf..

[23] Schelling X., Torres-Ronda L. (2016). Accelerometer load profiles for basketball-specific drills in elite players. J. Sports Sci. Med..

[24] Fox J.L., Stanton R., Scanlan A.T. (2018). A comparison of training and competition demands in semiprofessional male basketball players. Res. Q. Exerc. Sport..

[25] Foster C. (1998). Monitoring training in athletes with reference to overtraining syndrome. Med. Sci. Sports Exerc..

[26] Foster C., Florhaug J.A., Franklin J., Gottschall L., Hrovatin L.A., Parker S., Doleshal P., Dodge C. (2001). A new approach to monitoring exercise training. J. Strength Cond. Res..

[27] Alexiou H., Coutts A.J. (2008). A comparison of methods used for quantifying internal training load in women soccer players. Int. J. Sports Physiol. Perf..

[28] Coutts A.J., Reaburn P. (2008). Monitoring changes in rugby league players’ perceived stress and recovery during intensified training. Percept. Mot. Skills.

[29] Klusemann M.J., Pyne D.B., Hopkins W.G., Drinkwater E.J. (2013). Activity profiles and demands of seasonal and tournament basketball competition. Int. J. Sports Physiol. Perf..

[30] Moreira A., McGuigan M.R., Arruda A.F., Freitas C.G., Aoki M.S. (2012). Monitoring internal load parameters during simulated and official basketball matches. J. Strength Cond. Res..

[31] Montgomery P.G., Pyne D.B., Minahan C.L. (2010). The physical and physiological demands of basketball training and competition. Int. J. Sports Physiol. Perf..

[32] Scanlan A.T., Wen N., Tucker P.S., Dalbo V.J. (2014). The relationships between internal and external training load models during basketball training. J. Strength Cond. Res..

[33] Dellal A., Keller D., Carling C., Chaouachi A., Wong del P., Chamari K. (2010). Physiologic effects of directional changes in intermittent exercise in soccer players. J. Sterngth Cond Res..

[34] McInnes S., Carlson J.S., Jones C.J., McKenna M.J. (1995). The physiological load imposed on basketball players during competition. J. Sports Sci..

[35] Dawes J.J., Spiteri T. (2016). Relationship between pre-season testing performance and playing time among NCAA DII basketball players. Sports Exerc. Med..

[36] Hoffman J.R., Tenenbaum G., Maresh C.M., Kraemer W.J. (1996). Relationship between athletic performance tests and playing time in elite college basketball players. J. Strength Cond. Res..

[37] McGill S.M., Andersen J.T., Horne A.D. (2012). Predicting performance and injury resilience from movement quality and fitness scores in a basketball team over 2 years. J. Strength Cond. Res..

[38] Chatzinikolaou A., Draganidis D., Avloniti A., Karipidis A., Jamurtas A.Z., Skevaki C.L., Tsoukas D., Sovatzidis A., Theodorou A., Kambas A. (2014). The microcycle of inflammation and performance changes after a basketball match. J. Sports Sci..

[39] Buchheit M. (2015). Sensitivity of monthly heart rate and psychometric measures for monitoring physical performance in highly trained young handball players. Int. J. Sports Med..

[40] Coutts A.J., Slattery K.M., Wallace L.K. (2007). Practical tests for monitoring performance, fatigue and recovery in triathletes. J. Sci. Med. Sport..

[41] Kenttä G., Hassmén P. (1998). Overtraining and recovery. Sports Med..

[42] Gallo T.F., Cormack S.J., Gabbett T.J., Lorenzen C.H. (2017). Self-reported wellness profiles of professional Australian football players during the competition phase of the season. J. Strength Cond. Res..

[43] Komi P.V. (2000). Stretch-shortening cycle: A powerful model to study normal and fatigued muscle. J. Biomech..

[44] Nicol C., Avela J., Komi P.V. (2006). The stretch-shortening cycle. Sports Med..

[45] Legg J., Pyne D.B., Semple S., Ball N. (2017). Variability of jump kinetics related to training load in elite female basketball. Sports.

[46] Markwick W. (2015). Training Load Quantification in Professional Australian Basketball and the Use of the Reactive Strength Index as a Monitoring Tool. Master’s Thesis.

[47] Claudino J.G., Cronin J., Mezencio B., McMaster D.T., McGuigan M., Tricoli V., Amadio A.C., Serrao J.C. (2017). The countermovement jump to monitor neuromuscular status: A meta-analysis. J. Sci. Med. Sport..

[48] Ronglan L., Raastad T., Børgesen A. (2006). Neuromuscular fatigue and recovery in elite female handball players. Scand. J. Med. Sci. Sports..

[49] Gibson N.E., Boyd A.J., Murray A.M. (2016). Countermovement jump is not affected during final competition preparation periods in elite rugby sevens players. J. Strength Cond. Res..

[50] Gathercole R., Sporer B., Stellingwerff T., Sleivert G. (2015). Alternative countermovement-jump analysis to quantify acute neuromuscular fatigue. Int. J. Sports Physiol. Perform..

[51] Spiteri T., Nimphius S., Wolski A., Bird S. (2013). Monitoring neuromuscular fatigue in female basketball players across training and game performance. J. Aust. Strength Cond..

[52] Spiteri T., Nimphius S., Hart N.H., Specos C., Sheppard J.M., Newton R.U. (2014). Contribution of strength characteristics to change of direction and agility performance in female basketball athletes. J. Strength Cond. Res..

[53] Achten J., Jeukendrup A.E. (2003). Heart rate monitoring. Sports Med..

[54] Bosquet L., Merkari S., Arvisais D., Aubert A.E. (2008). Is heart rate a convenient tool to monitor over-reaching? A systematic review of the literature. Br. J. Sports Med..

[55] Dressendorfer R.H., Wade C.E., Scaff J.H. (1985). Increased morning heart rate in runners: A valid sign of overtraining?. Phys. Sportsmed..

[56] Fry A.C., Kraemer W.J., van Borselen F., Lynch J.M., Marsit J.L., Roy E.P., Triplett N.T., Knuttgen H.G. (1994). Performance decrements with high-intensity. Med. Sci. Sports Exerc..

[57] Fry R.W., Morton A.R., Keast D. (1991). Overtraining in athletes. Sports Med..

[58] Al Haddad H., Laursen P.B., Chollet D., Ahmaidi S., Bucheit M. (2011). Reliability of resting and postexercise heart rate measures. Int. J. Sports Med..

[59] Esco M.R., Flatt A.A. (2014). Ultra-short-term heart rate variability indexes at rest and post-exercise in athletes: evaluating the agreement with accepted recommendations. J. Sports Sci. Med..

[60] Chen J.L., Yeh D.P., Lee J.P., Huang C.Y., Lee S.D., Chen C.C., Kuo T.B., Kao C.L., Kuo C.H. (2011). Parasympathetic nervous activity mirrors recovery status in weightlifting performance after training. J. Strength Cond. Res..

[61] Atlaoui D., Pichot V., Lacoste L., Barale F., Lacour J.R., Chatard J.C. (2007). Heart rate variability, training variation and performance in elite swimmers. Int. J. Sports Med..

[62] Pichot V., Roche F., Gaspoz J.M., Barthelemy J.C. (2000). Relation between heart rate variability and training load in middle-distance runners. Med. Sci. Sports Exerc..

[63] Haff G.G., Triplett N.T. (2015). Essentials of Strength Training and Conditioning.

[64] Hug M., Mullis P.E., Vogt M., Ventura N., Hoppeler H. (2003). Training modalities: Over-reaching and over-training in athletes, including a study of the role of hormones. Best Pract. Res. Clin. Endocrinol. Metab..

[65] Häkkinen K., Pakarinen A., Alen M., Kauhanen H., Komi P.V. (1988). Daily hormonal and neuromuscular responses to intensive strength training in 1 week. Int. J. Sports Med..

[66] Häkkinen K., Pakarinen A., Alen M., Kauhanen H., Komi P.V. (1987). Relationships between training volume, physical performance capacity, and serum hormone concentrations during prolonged training in elite weight lifters. Int. J. Sports Med..

[67] Maron M.B., Horvath S.M., Wilkerson J.E. (1977). Blood biochemical alterations during recovery from competitive marathon running. Eur. J. Appl. Physiol..

[68] Cormack S.J., Newton R.U., McGuigan M. (2008). Neuromuscular and endocrine responses of elite players to an Australian rules football match. Int. J. Sports Physiol. Perform..

[69] Banfi G., Marinelli M., Roi G.S., Agape V. (1993). Usefulness of free testosterone/cortisol ratio during a season of elite speed skating athletes. Int. J. Sports Med..

[70] Schelling X., Calleja-Gonzalez J., Torres-Rinda L., Terrados N. (2015). Using testosterone and cortisol as biomarker for training individualization in elite basketball: A 4-year follow-up study. J. Strength Cond. Res..

[71] Hoffman J.R., Epstein S., Yarom Y., Einbinder M. (1999). Hormonal and biochemical changes in elite basketball players during a 4-week training camp. J. Strength Cond. Res..

[72] Montgomery P.G., Pyne D.B., Cox A.J., Hopkins W.G., Minahan C.L., Hunt P.H. (2008). Muscle damage, inflammation, and recovery interventions during a 3-day basketball tournament. Eur. J. Sports Sci..

[73] Coutts A., Reaburn P., Piva T.J., Murphy A. (2007). Changes in selected biochemical, muscular strength, power, and endurance measures during deliberate overreaching and tapering in rugby league players. Int. J. Sports Med..

[74] Kargotich S., Keast D., Goodman C., Morton A.R. (2007). Monitoring 6 weeks of progressive endurance training with plasma glutamine. Int. J. Sports Med..

[75] Hartmann U., Mester J. (2000). Training and overtraining markers in selected sport events. Med. Sci. Sports Exerc..

[76] Windt J., Gabbett T.J., Ferris D., Khan K.M. (2016). Training load—Injury paradox: Is greater preseason participation associated with lower in-season injury risk in elite rugby league players?. Br. J. Sports Med..

[77] Hägglund M., Walden M., Magnusson H., Kristenson K., Bengtsson H., Ekstand J. (2013). Injuries affect team performance negatively in professional football: An 11-year follow-up of the UEFA Champions League injury study. Br. J. Sports Med..

[78] Murray N.B., Gabbett T.J., Chamari K. (2014). Effect of different between-match recovery times on the activity profiles and injury rates of national rugby league players. J. Strength Cond. Res..

[79] Dellal A., Lago-Penas C., Rey E., Chamari K., Orhant E. (2013). The effects of a congested fixture period on physical performance, technical activity and injury rate during matches in a professional soccer team. Br. J. Sports Med..

